# When the seeds sprout, the hornbills hatch: understanding the traditional ecological knowledge of the Ibans of Brunei Darussalam on hornbills

**DOI:** 10.1186/s13002-019-0325-0

**Published:** 2019-09-02

**Authors:** F. Merlin Franco, Misa Juliana Minggu

**Affiliations:** 1 Institute of Asian Studies, Universiti Brunei Darussalam, Jalan Tungku Link, Gadong, BE1410 Brunei Darussalam; 2 Institute of Asian Studies, Universiti Brunei Darussalam, Jalan Tungku Link, Gadong, BE1410 Brunei Darussalam

**Keywords:** Ethnoecology, Local knowledge, Indigenous knowledge, Bucerotidae, Borneo

## Abstract

**Background:**

Hornbills are known to play an important role in rainforests as agents of seed dispersal. Decades of scientific research has led to a vital body of knowledge on hornbill taxonomy, ecology, distribution, and conservation status. However, the traditional ecological knowledge (TEK) that local people possess on hornbills has largely been underexplored. In 2018, we collaborated with the Iban people of Temburong, Brunei Darussalam, to study their TEK on hornbills.

**Method:**

We collaborated with the members of the Iban community from four longhouses and four villages in Temburong, Brunei Darussalam. Our study adopts a qualitative approach; we used detailed semi-directive interviews and brief semi-structured interviews to gather data. The semi-directive interviews documented the TEK related to Hornbills in detail while the brief semi-structured interviews assessed the current status of TEK in the age group of 18–40 years.

**Results:**

The results show that the Iban ethnotaxonomy recognises seven folk species of hornbills, with Asian Black Hornbill (*Anthracoceros malayanus*) and Oriental Pied Hornbill (*Anthracoceros albirostris*) considered as a single folk species. The Iban TEK on diet and reproductive behaviour of hornbills complement existing scientific records, with the Iban TEK providing additional locale-specific information on the dietary preferences, abundance and conservation threats. However, the average Iban member has lost much of this TEK, and it is the subsistence hunters and agriculturists who have conserved it.

**Conclusion:**

There is an urgent need for encouraging transmission of knowledge from the hunters and agriculturists to others through ecotourism and conservation ventures. Our study adds further support to the understanding that the TEK of local communities is an important source of locale-specific knowledge on species of high conservation value such as hornbills.

## Introduction

Hornbills are large birds belonging to the Bucerotidae family and are known to possess several unique traits in the avian world such as cooperative breeding, female self-incarceration during nesting and mostly monogamous pairing [1, 2]. Of the 59 extant species of hornbills, 31 are found in Asia [3–5]. Largely frugivores by diet, hornbills play an important role in dispersing seeds of fruits above 2 cm in diameter in the tropical forests of Africa and Asia [4, 6–8], ensuring the survival and regeneration of forest ecosystems. Thus, they are fondly referred to as ‘farmers of the forest’ [9]. In human farming communities, the favourable season of marriages generally follow the harvest when there is plenty of food available. For the farmers of the forest too, availability of fruits is one of the two crucial factors for breeding [10, 11], the other factor being availability of tall trees with cavities for nesting [12]. Naturally, primary lowland rainforests that are rich in tall growing and fruit-bearing trees form the principal habitats of hornbills [13]. However, logged forests could also serve as refuges to hornbills, making them habitats worth conserving in contemporary times [14–18].

Hornbills are slow-breeding species requiring large tracts of forests to survive. The estimated offspring production rate of Rhinoceros Hornbills breeding once in 1.5 years is 2.0625 per 10 birds in the population [19]. For the survival of our remaining rainforests, it is critical that these offspring grow into maturity and fulfil their ecological role. However, forest loss, logging and hunting pose severe challenges to the survival of extant hornbill species [20, 21]. The island of Borneo where Brunei Darussalam is situated is home to eight species of hornbills: *Anthracoceros malayanus* (Asian Black Hornbill), *Anthracoceros albirostris* (oriental pied hornbill), *Anorrhinus galeritus* (Bushy-crested Hornbill), *Berenicornis comatus* (White-crested Hornbill/White-crowned hornbill), *Buceros rhinoceros* (Rhinoceros Hornbill), *Rhabdotorrhinus corrugatus* (Wrinkled Hornbill), *Rhinoplax vigil* (Helmeted Hornbill) and *Rhyticeros undulatus* (Wreathed Hornbill). All these species except *A. albirostris* are of immediate concern from the conservation point of view [22].

In Borneo, hunting of hornbills by various communities is one of the reasons for the rapid decline in their populations [23]. Feathers of Helmeted, Rhinoceros and Asian Black Hornbills are used by Iban and Orang Ulu men and women as adornments during traditional dances performed during festivals such as the Gawai Kenyalang, while their casque is used to make earrings [19, 21]. Researchers have questioned if hornbills, culture and hunting practices can co-exist [19]. Loss of forests and cultural loss have happened on parallel fronts in Borneo, leading to either downscaling or abandoning of festivals such as Gawai [24]. However, there is no decline in hunting, and the advent of commercial hunting and shotguns have only aggravated the rate of species loss due to hunting [23, 25]. A report by Hadiprakarsa et al. [26] shows that around 6000 helmeted hornbills are hunted per year in the Indonesian Borneo. Bennett et al. [23], while discussing the magnitude of animals hunted by loggers, commented that the loggers who were adept in hunting were Ibans from traditional hunting backgrounds, offering a glimpse of the traditional ecological knowledge (TEK) possessed by the Iban hunters [25]. Decades of hard work by researchers have ensured that we have quality scientific data on the hornbills of the Sundaland hotspot [10, 11, 13, 16, 19, 20, 23, 26–28]. The cultural importance of hornbills to Borneo’s indigenous communities such as Ibans has also been fairly documented [24, 29–32]. However, there is dearth of studies on the TEK on hornbills of Borneo and nearby regions. The documentation of Iban TEK on wild birds in Sarawak [33] and the exceptional work on the TEK of Filipino communities [34] are noteworthy examples of the few studies that exist. This observation is in line with the analysis of scientific publications for 25 years by Brook and McLachlan [35] which shows that papers reporting TEK are lacking.

TEK can provide information on the temporal and spatial distribution patterns of different species that could complement scientific data, and thus aid in fostering biodiversity conservation and management [36–38]. The discovery of the snub-nosed monkey exclusively on the basis of the TEK provided by hunters of Kachin, Myanmar [39], the natural resource management techniques of Maori and Dusun people [40], conservation of Orangutans through taboos [41] and weather forecasting by the Kenyah of Borneo [42] are examples of the contemporary relevance of TEK. TEK is not a product of trial and error, but a result of long-term observation of their environment using logic and reasoning that is usually associated with formal science [43]. Given that hornbills are an important group of birds both for the ecosystem as well as for local cultures, it is important to understand the birds from both scientific and traditional perspectives. Hence, we conceived this study to understand the TEK of the Iban community of Temburong in Brunei Darussalam on their hornbills and the cultural importance accorded to them. The cultural importance of hornbills will be reported elsewhere.

## Methods

Our study adopts a qualitative approach to understand the Iban TEK on hornbills. Studies in conservation and ecology rely heavily on quantitative methods. However, studies using qualitative approaches have much to offer to these fields by generating data that help in the ‘understanding of categories, processes, relationships and perceptions [44]. We used detailed semi-directive interviews and brief semi-structured interviews to gather data. The semi-directive interviews were meant to document the TEK related to hornbills in detail and thus formed the core of the study; the brief semi-structured interviews were only meant to quickly assess the current status of TEK in the age group of 18–40 years. Semi-directive interviews have been proven to be useful in documenting TEK [45]. The themes for semi-directive interviews were adapted from Ternes et al. [46]. We use the definition of TEK as *all types of knowledge about the environment derived from the experience and traditions of a particular group of people* [47]. We consulted Moon et al. [48] to ensure that the resulting study would conform to the standards of quality expected from qualitative researches in ecology and conservation.

### Study area

We collaborated with the members of the Iban community from four longhouses and four villages in Temburong, Brunei Darussalam. Temburong is the second largest district in Brunei Darussalam with a total area of 1306 km^2^ [49]. In 2010, the population of the district was 9193 individuals, of which the population of Ibans was made up of 2092 people [49]. Owing to the extremely small population, it is possible to identify prominent personalities by linking location and demographic data. Hence, to ensure anonymity of our informants, we are withholding the exact localities where the study was conducted. To the south of the district is the Batu Apoi Forest Reserve that encompasses the Ulu Temburong National Park. The vegetation here is largely made up of mixed Dipterocarp forests rich in biodiversity and endemism [50, 51]. There is no dry season, and the precipitation could reach up to 4000 mm [52]. The northern end of the district is the Brunei Bay, close to which are the Mangrove forests characterised by the presence of large trees growing up to 20–40 cm in diameter [53].

### The Ibans

The Ibans are known for their warfare skills, traditional knowledge about the environment and the Gawai Kenyalang festival that features the Rhinoceros Hornbill [24, 54]**.** They are believed to have originated in West Kalimantan in Borneo before mass migrations took place between the 16th and the 19th centuries that brought them to Sarawak and eventually to Brunei [54, 55]. The Ibans who had migrated to Brunei after the discovery of oil do not qualify for the ‘Yellow ICs’ that provide full citizenship privileges, as per the Brunei Nationality Act 1961. The Ibans are not legally considered as indigenous in Brunei unlike Sarawak and Kalimantan [56]. However, for the purpose of this study, we apply ‘indigenous’ in the context of the island of Borneo. Traditionally, the Ibans practised swidden agriculture and subsistence hunting. They speak the Iban language which belongs to Ibanic subcategory of the Malayic group [55]. Contrary to popular belief that the Iban language in Brunei is being rapidly lost due to acculturation, studies show that the language still maintains high vitality [55, 57].

### Sampling, data collection and analysis

We conducted both semi-directive and semi-structured interviews with two different set of participants in August and December 2018. For the semi-directive interviews, we collaborated with 18 knowledgeable elders from the community who expressed willingness to participate in the study (male = 10; female = 08). We refer to this group of participants as ‘elders’. These elders were identified purposively using a lone criteria—their reputation in the community for knowledge on hornbills. Among the elders recommended by community members were two men who frequently ventured into the forests for subsistence hunting and one female who was involved in agriculture during her young age. We refer to these three elders as ‘specialists’. As the second author is a member of the Iban community and spoke the same language, establishing rapport with the elders was easier. There were no fixed questionnaires or time limits, but the discussions were guided to follow leads adapted from Ternes et al. [46]. Discussion topics were aimed at collecting the following details: (1) demographic particulars such as age, gender, longhouse, occupation and educational level; (2) ethnotaxonomy of hornbills; (3) distribution of hornbills in the Temburong region; (4) trophic ecology—dietary behaviour and level occupied in food chain; (5) reproductive aspects; (6) human uses of hornbills and their parts; and (7) abundance, trends and threats. Following the semi-directive interviews, 30 participants selected through snowballing (male = 14, female = 16) were interviewed using a semi-structured questionnaire. Apart from demographic particulars, there were questions eliciting the following information: (1) the different hornbill species recognised by the Iban people, (2) Iban names for the hornbills and (3) localities where they can be sighted. There were also other questions on the cultural importance of hornbills, the results of which would be reported elsewhere. The respondents were members of the younger generation in the age group of 18 to 40 years, recommended by members of the longhouses and villages we visited. These members were recommended on the basis of their availability for interview. As most of the members of the younger generation have migrated out of Temburong, finding possible respondents was a cumbersome task. Semi-directive interviews were audio-recorded and later transcribed into the field notes. Semi-structured interviews were recorded on paper. Data was analysed as per the themes and verified with the participants of the semi-directive interviews. The socio-economic profiles of the participants of the open-ended and semi-structured interviews are provided in Tables 1 and 2.

**Table 1:** Demographic particulars of participants of semi-directive interviews

Particulars		Number of participants
Age class	41–50	2
	51–60	2
	61 and above	14
Gender	Male	10
	Female	8
Occupation	Home keeper	6
	Teacher	1
	Retired from government	1
	Retired teacher	1
	Retired from formal sector	1
	Retired from informal sector	4
	Undisclosed	4
Educational level	No formal education	3
	Below secondary	11
	Secondary school	3
	Undergraduate	1

**Table 2:** Demographic particulars of participants of semi-structured interviews

Particulars		Number of respondents
Age Class	18–30	18
	31–40	12
Gender	Male	14
	Female	16
Occupation	Student	11
	Home keeper	5
	Self-employed	1
	Construction	2
	Clerical & private sector	4
	Driver	1
	Government	6
Education	Secondary level	18
	Tertiary level	12

## Results

Ibans recognise seven folk species of hornbills. In the elders’ group, of the ten men interviewed, only two were able to name and identify the seven folk species. Of the eight female elders, only one was able to name and identify the seven folk species. The rest of the elders’ group were mostly able to name and identify only three folk species—*kenyalang* (*Buceros rhinoceros*), *kekalau* (*Anorrhinus galeritus*) and *bruie* (*Anthracoceros* spp.). All participants were able to provide information related to habitat and dietary preference of the hornbill species they had listed and identified. Data from the semi-structured interviews with the younger generation show that most of the younger respondents were not familiar with the folk species except *bruie* and *kenyalang*. Fourteen respondents listed only *bruie* and *kenyalang* as the species known to the Iban people; one respondent listed *tajak*, *bruie* and *kenyalang*; 11 listed *kenyalang* alone; and four listed none. We did not find any noteworthy patterns related to age, education or gender here. Most respondents could not provide precise localities where the hornbills can be sighted. ‘Jungle, hill and tall trees’ were named as locations to sight instead. Only three respondents named places such as Belalong, Biang and Selapon where hornbills can be spotted. Twenty-two respondents did not have an answer. Again, we do not find any significant patterns related to age, education or gender in these responses.

### Iban TEK on Hornbills

#### Ethnotaxonomy and nomenclature

Iban ethnotaxonomy recognises seven species of hornbills with Asian Black Hornbill and Oriental Pied Hornbill considered as one folk species identified by the name *bruie* (Table 3). Four participants identified the Asian Black Hornbill with the name *likap gagak* (crow hornbill). Participants were able to distinguish both the species on the basis of their morphology and habitat preferences when asked. It is inferred from the interviews that Iban ethnotaxonomy considers both Asian Black Hornbill and Oriental Pied Hornbill as subspecies of the folk species *bruie*. The Iban ethnotaxonomic system also uses hornbill names to identify places in Temburong. We collected two such names: *Sungai Kenyalang* and *Rantau Tajak*. *Sungai Kenyalang* (*sungai* = river, *kenyalang* = hornbill) is a tributary of the River Sibut. However, respondents could not explain the reasoning behind the name of the river. Likewise, we could not record the reasoning behind Rantau Tajak (*rantau* = rocky part of a river; *tajak* = helmeted hornbill) as the respondents could not explain it. The Iban people are known to name localities after significant events. For instance, *Sungai Sawa* (*sungai* = river; *sawa* = python) is named after an incident where a community member was caught by a python while collecting leaves for food wraps. Hence, it is possible that these spots have been named for events connected to the *kenyalang* and *tajak* respectively. The Iban ethnotaxonomy of place categorises Temburong into two: the *ulu* or the upstream of the river and the *ili* or the downstream. Belalong, Ulu Temburong National Park (UTNP), and nearby areas such as Batang Duri, Sumbiling, etc., form parts of *ulu* while Bangar, Labu, etc., are considered as *ili*.

**Table 3:** Summary of Iban TEK on hornbills

Scientific name	English name	Local names	Diet (Iban TEK)	Habitat and nesting (Iban TEK)	Conservation status	
					Local (Iban TEK)	Global (IUCN 2019)*
*Anthracoceros malayanus* (Raffles, 1822)	Asian Black Hornbill	*Likap gagak (M, D)*; *Bruie (I)*; *Alau babi (M)*	Fruits: *Dacryodes rostrata, Canarium odontophyllum*; *Ficus* spp., *Myristica* spp. Insects: cockroaches and lizards	Jungles closer to human settlements and human influenced habitats; nests on tall trees	Increasing population	Decreasing population (vulnerable)
*Anorrhinus galeritus* (Temminck, 1831)	Bushy-crested Hornbill	*Kekalau (I, M)*	Fruits: *Dacryodes rostrata*, *Canarium odontophyllum*; *Ficus* spp., *Myristica* spp. Insects: cockroaches and lizards	Jungles closer to human settlements and human influenced habitats; nests on tall trees	Increasing population	Decreasing (near threatened)
*Rhinoplax vigil* (J.R.Forster, 1781)	Helmeted Hornbill	*Tajai/Tajak (I)*; *Lantudon (M)*	Fruits: *Dacryodes rostrata, Canarium odontophyllum*; *Ficus* spp., *Myristica* spp. *Skinks*, *snakes*	Mixed dipterocarp forests; nests on tall lone trees	Increasing population	Decreasing (critically endangered)
*Anthracoceros albirostris* (Shaw, 1808)	Oriental Pied Hornbill	*Bruie (I)*; *Alau Pedada (M)*	Fruits: *Dacryodes rostrata*, *Canarium odontophyllum*; *Ficus* spp., *Myristica* spp.	Mangroves, swamp forests and forests close to mangroves; nests on relatively shorter trees and hence eggs are prone to poaching by Sun Bear	Increasing population	Stable (least concern)
*Buceros rhinoceros* Linnaeus, 1758	Rhinoceros Hornbill	*Kenyalang (I)*; *Loklang Sangang(M)*	Fruits: *Dacryodes rostrata, Canarium odontophyllum*; *Ficus* spp., *Myristica* spp. Skinks, snakes	Mixed dipterocarp forests; nests on tall lone trees; prefers quiet localities	Increasing population	Vulnerable (decreasing)
*Berenicornis comatus* (Raffles, 1822)	White-crested Hornbill/White-crowned Hornbill	*Sentuku (I,M)*	Fruits: *Dacryodes rostrata, Canarium odontophyllum*; *Ficus* spp., *Myristica* spp.	Swamp forests and mangroves; nests on tall trees in the habitat	Increasing population	Decreasing (endangered)
*Rhyticeros undulatus* (Shaw, 1811)	Wreathed Hornbill	*Undan/Kekuik (I), Alau Sangoh (M)*	Fruits: *Dacryodes rostrata*, *Canarium odontophyllum*; *Ficus* spp*.*, *Myristica* spp. Fishes, frogs including Giant River Frogs (*Limnonectes leporinus*)	Mixed dipterocarp and montane forests; riverine habitats; nests on tall trees; prefers quiet localities	Increasing population	Decreasing (vulnerable)
*Rhabdotorrhinus corrugatus* (Temminck, 1832)	Wrinkled Hornbill	*Kejakoh (I)*; *Alau buloh (M)*	Fruits: *Dacryodes rostrata*, *Canarium odontophyllum*; *Ficus* spp., *Myristica* spp., frogs including Giant River Frogs (*Limnonectes leporinus*)	Mixed dipterocarp and montane forests; nests on tall trees; prefers quiet localities	Increasing population	Decreasing (endangered)

*Source: [22]

#### Distribution and trophic ecology

**Fig. 1:**
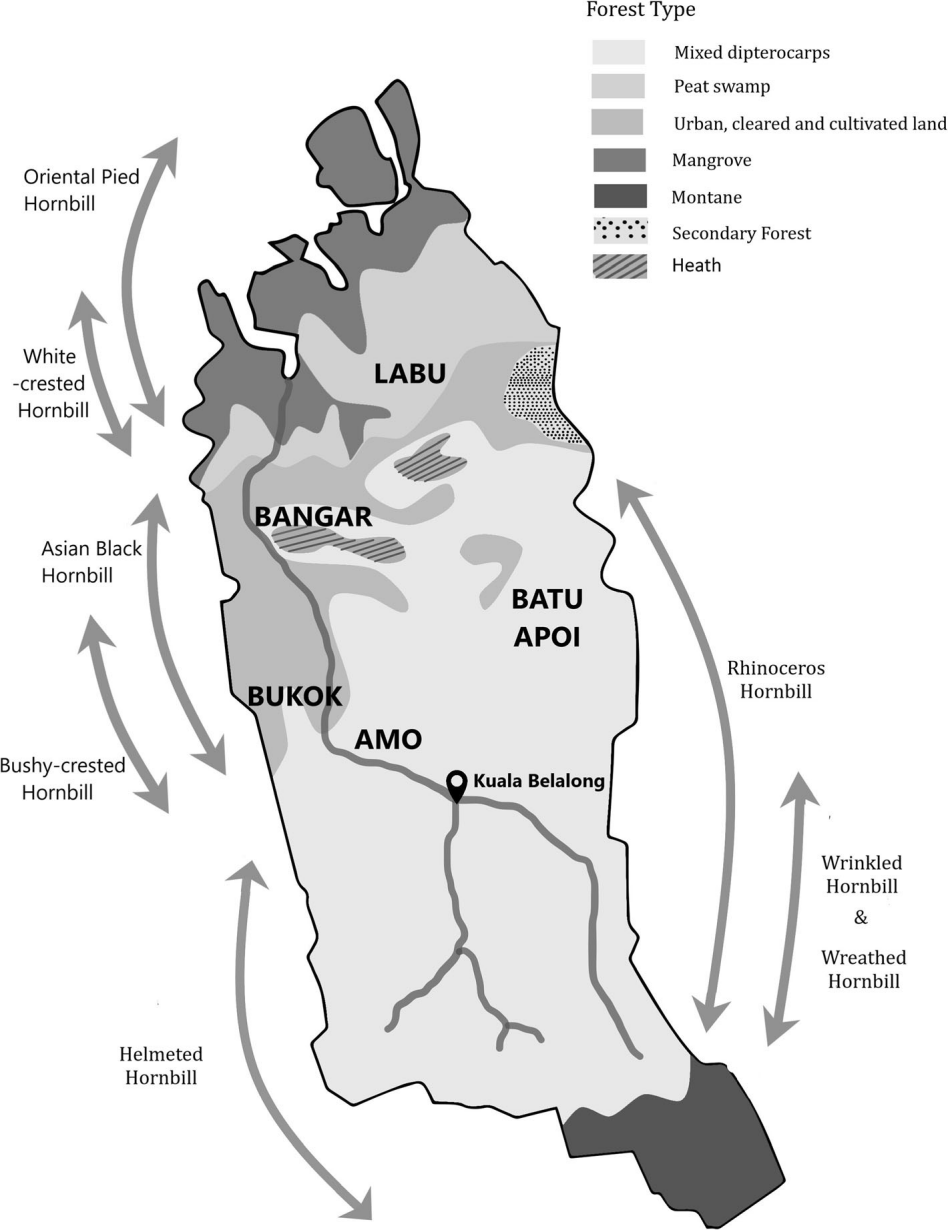
Map showing the distribution of hornbill species as per the Iban TEK

The elders were able to identify the various habitats preferred by the hornbill folk species they had identified (Fig. 1). However, they also confirmed that except the Helmeted Hornbill, all other species can also be sighted in the proximity of human settlements during the fruiting season of *kembayau* (*Dacryodes rostrata* (Blume) H.J.Lam), durians (*Durio* spp.) *dabai* (*Canarium odontophyllum* Miq.) and *buah kara* (*Ficus* spp.). In addition to these fruits, the Bushy-crested Hornbills also feed on cockroaches and lizards which are abundant in human settlements. However, these hornbills do not nest in the trees close to human settlements and return to their respective habitats in Temburong after feeding.

**Fig. 2:**
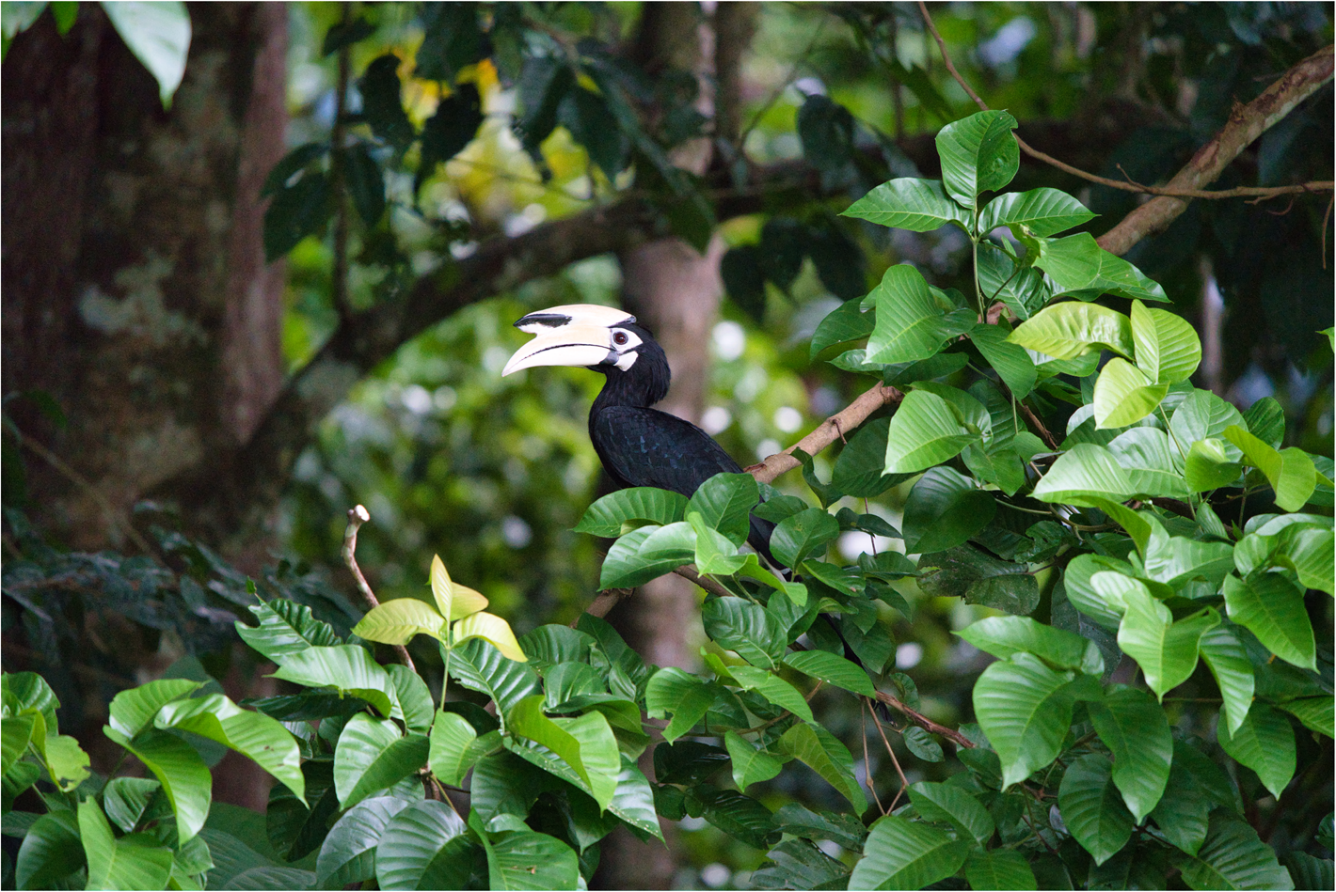
Oriental Pied Hornbill. Credits: F. Merlin Franco

According to Iban TEK, the Oriental Pied Hornbills (Fig. 2), which is considered as one of the sub-species of the folk species *bruie*, is found mostly in wetlands such as mangroves, swamps and the nearby jungles, while the Asian Black Hornbill prefers habitats other than wetlands. According to the specialists we spoke to, the habitat preferences of both these species do not overlap. The Wreathed Hornbill consumes both fig fruits and fishes and is hence found in the riverine habitat. The Wrinkled Hornbills hunt the Giant River Frog which is also hunted by the Ibans. However, the Iban people avoid competing with the hornbills for frogs. They relate to this using the analogy of the jungle cat and Mouse Deer: if the jungle cat spots the Mouse Deer before an Iban, then it has its right to prey, and vice versa.

Elders reported that hornbills regurgitate the seeds after swallowing the fleshy part of fruits*.* They also defecate the seeds after digesting, thus helping in seed dispersal. Thus, trees frequented by hornbills are characterised by the presence of numerous saplings on the floor beneath. Large hornbill species such as Rhinoceros and Helmeted Hornbills feed on small snakes and skinks. Two of the three specialists reported a hierarchy among the hornbills existing at the dietary and spatial levels. The Rhinoceros Hornbill occupies the top position in the hierarchy, immediately followed by the Helmeted Hornbill. Next in the order are the Wrinkled and Wreathed hornbills, competing for the same diet and space and hence can be rarely found in the same locality. All other species occupy the lowest level of the hierarchy where no competition exists, due to smaller body sizes. When a fig tree fruits, the Rhinoceros Hornbill dominates the scene with no other species permitted to feed on the figs. The next bird to dominate the scene is the Helmeted Hornbill. Other species are forced to wait until the dominant birds fly away.

#### Reproductive aspects

Elders quoted fruiting season of wild and semi-domesticated trees as the breeding season of hornbills. This period, which coincides with the months of July to August, is considered as *musin nugal*, the period of paddy cultivation. However, if *kembayau* (*Dacryodes rostrata*)*, dabai* (*Canarium odontophyllum*) and *buah kara* (fruit of *Ficus* spp.) fruit twice in a year, then there would be two nesting seasons too. Hornbills other than Oriental Pied and White-crested prefer tall trees for nesting purposes. Examples of such tree species are *pengerawan* (*Hopea mengarawan* Miq.)*, tapang* (*Koompassia excelsa* (Becc.) Taub.), *tekam****/****merawan* (*Hopea* spp.), *kapur* (*Dryobalanops* spp.)*, meranti* (*Shorea* spp.) and *selangan batu* (*Shorea* spp.)*.* Though the tree species are same, the Rhinoceros and Helmeted hornbills prefer to build their nest on lone trees, avoiding clustered ones. This is one of the reasons why *Koompassia excelsa* is a preferred tree species for both these hornbill species, as the emergent tree offers a safe habitat. Elders also reported nest-site fidelity as the norm in all hornbill species, as long as the burrow remains hospitable. They also stated that larger species of hornbills such as *kenyalang*, *tajak*, *kekuik* and *bruie* lay only one or two eggs. A specialist reported that in *kekalau*, the clutch size is more than three, and all of them reach maturity and fly off from the nest. The same specialist also reported that for Bushy-crested Hornbills, the flock feeds the mother and chicks. The specialist also claimed that rarely a male bird other than the actual partner could feed the female bird while nesting. Such act of ‘cheating’ is reported to occur in all species except the socially breeding Bushy-crested Hornbill. Acts of cheating do not go unnoticed as the male partner could guess it from the reduced appetite of the female. The male partner then deserts the female, leaving it to perish in the nest.

The Iban people recognise four distinct stages in the breeding of hornbills, from laying of eggs to hatching. These stages are identified from the seedlings that sprout on the forest floor adjacent to the nesting tree:

i. Presence of seeds: Eggs are laid and the mother starts incubating it. The seeds expectorated are found on the forest floor. This stage is referred to as *buah sapi* (*buah* = fruit, *sapi* = feeding).
ii. When the seedlings emerge and show two leaves, the eggs hatch. This stage is called as *anak kayu sapi* (*anak* = child; *kayu* = tree). At this stage, it is impossible to confirm the botanical identity of the seedling.
iii. When the seedlings show four leaves, the chicks show feathers. The botanical identity of the seedling cannot be determined at this stage.
iv. The seedling grows further, and it is now possible to determine its identity. It is time for the mother to emerge from the nest. The young birds would emerge from the nest a week later.

#### Abundance, trends and threats

According to the Iban TEK, the population of hornbills has been increasing in Temburong. The elders attribute this to two important factors: (i) Following the rebellion of 1962, guns were banned in Temburong and, as a result, people have stopped hunting hornbills. (ii) The massive deforestation and logging activities in the neighbouring Malaysian state of Sarawak has led to the migration of birds to Temburong where there has been little loss of forests. Three participants also claimed that sightings of Helmeted and Wreathed hornbills in urban areas have declined due to the changes brought by the development of lands for housing and roads, and the noisy ambience. However, there has been no decline in sightings of the Rhinoceros Hornbill in human habitats with sightings increasing during wild fruit seasons especially in the *ulu* (interior) areas such as Belalong and Sumbiling.

### Current status of Iban TEK

In the elders’ group, we did not find a correlation between formal education and lack of TEK; participation in traditional hunting activities and participation in traditional agricultural activities are the two factors that explain the difference in the status of TEK. The two men who were exceptionally knowledgeable about the ethnoecology of hornbills had participated in hunting trips with their parents and grandparents where they had the opportunity to acquire TEK related to hornbills. They continue to frequent the jungle for hunting non-protected species for subsistence purposes even today. The female specialist was involved in rice cultivation during her younger ages which offered her opportunities to observe the hornbills in the field, as well as participate in informal learning from fellow community members, especially elderly women. The rest of the elders’ group were mostly able to name and identify only three folk species—*kenyalang* (*Buceros rhinoceros*), *kekalau* (*Anorrhinus galeritus*) and *bruie* (*Anthracoceros* spp.). These species are commonly encountered in the human-inhabited areas of Temburong. All female elders identified abandoning of rice farming as the major reason for lack of opportunities to acquire TEK, while men identified the abandoning of the practice of venturing into the jungle for hunting and fishing as the major reason. Amongst the younger generation too, we did not find any noteworthy patterns related to age, education or gender. Most respondents could not name the precise localities where the hornbills can be sighted, with ‘jungle, hill, and tall trees’ named as locations to sight instead. Only three respondents provided places such as Belalong, Biang and Selapon where hornbills can be spotted. Fifteen respondents did not have an answer. Again, we do not find any noteworthy patterns related to age, education or gender in these responses.

The elders identified five major factors that drive TEK loss in the Iban community as described below. The results of the study show that abandoning of traditional hunting and shift in occupational preference are the primary factors responsible for loss of TEK, while urban drift, formal education and lack of incentives play a secondary role.

i. Urban drift

The population of longhouse residents has decreased over the years as community members have moved to individual housing or have migrated temporarily to other districts to pursue higher education and employment opportunities. These youngsters visit longhouses only during important occasions and do not get enough opportunities to learn the rituals and Iban traditional knowledge.

ii. Abandoning of the practice of traditional hunting

Although hunting of protected animals is banned as per the Wildlife Protection Act of 1984, hunting and trapping of animals that are not protected by the Act occur for subsistence purposes. Being in a hunting party offers young Ibans to socialise with elders and acquire TEK on the forest. Younger members affected by urban drift lose this crucial opportunity. The women folk who are not involved in traditional agriculture acquire their TEK from spouses who return from hunting parties. Thus for the female members, opportunity to acquire TEK is lost when their spouse is not involved in the traditional practice. None of the respondents from the younger generation interviewed were involved in hunting or rice cultivation, lending credence to this claim.

iii. Shift in occupational preference from rice cultivation/hunters to salaried jobs

For young Ibans who secure employment in government and private sectors, there is no immediate need to acquire the traditional knowledge and skills associated with farming and hunting. The elders allude to this, citing the proliferation of supermarkets that have replaced the jungle as source of food for the younger generation. For the womenfolk, agricultural fields previously offered opportunities to observe hornbills and other birds in field conditions and also acquire and transmit relevant TEK. Shift in occupation preference deprives them the opportunity to be on the field, observe their ecosystem and discuss knowledge related to hornbills.

iv. Formal education

The introduction of compulsory formal education and free access to education is also cited as factor for the loss of TEK among the young Iban generation. Similar to occupational change, formal education has replaced the informal learning opportunities that are quintessential for TEK acquisition.

v. Lack of incentives to acquire TEK on hornbills

The Ibans are not a business community and thus lack the networks and capacity required for operating businesses such as ecotourism ventures that could have utilised the TEK. At present, the only employment opportunity in the tourism sector for them is to be a boat operator or a tour guide. The elders reported that the tour operators emphasise on formal ecological knowledge of Temburong TEK. We made efforts to contact a prominent tour guide from the community who refused to collaborate stating that “if you want to see hornbills, I can help you. But, if you want to know about Iban knowledge on hornbills or its cultural importance, you should go to Sarawak. There is nothing left here.”

## Discussion

### Iban TEK on hornbills

#### Ethnotaxonomy and nomenclature

Borneo is home to eight species of hornbills, and all eight species are found in Brunei Darussalam. However, our study shows that Iban ethnotaxonomy recognises only seven folk species viz., *bruie* (*Anthracoceros* spp.), *kekalau* (*Anorrhinus galeritus*), *tajak* (*Rhinoplax vigil*), *kenyalang* (*Buceros rhinoceros*), *sentuku* (*Berenicornis comatus*), *undan* (*Rhyticeros undulatus*) and *kejakoh* (*Rhabdotorrhinus corrugatus*). The term *likap gagak* (crow hornbill) used by some participants to identify the Asian Black Hornbill as a separate species is a borrowed knowledge. *Likap* is the name given to Oriental Pied Hornbills, and *gagak* for crows in the Kedayan language [58], and the epithets have been combined to denote the Asian Black Hornbill. Besides, the Ibans of the neighbouring Malaysian state of Sarawak also identify Asian Black Hornbills by the folk name *bruie* [33], further attesting the Iban origin of the term *bruie*. As reported under the trophic ecology section, Oriental Pied Hornbills and Asian Black Hornbills are considered as subspecies of the folk species *bruie*, with distinct habitat preferences. The former is found mostly in wetlands such as mangroves, swamps and the nearby jungles, while the latter prefers habitats other than wetlands. The recognition of both the species of *Anthracoceros* as subspecies of the folk species *bruie* is unique to the Iban ethnotaxonomic system. This recognition is also in contrast to the scientific knowledge that *A. malayanus* and *A. albirostris* are two different species, with a genetic distance of 0.032 (3.2%), which comfortably exceeds the threshold required for delineating species [59, 60]. Although we could not identify the exact reasons behind the practice of attaching hornbill names to certain locations (*Sungai Kenyalang* and *Rantau Tajak*), the names are TEK records connecting the hornbills to the locality.

#### Distribution and trophic ecology

With the exception of the Helmeted Hornbill, all other species can be sighted in the proximity of human settlements during the fruiting season of *kembayau* (*Dacryodes rostrata*), durians (*Durio* spp.), *dabai* (*Canarium odontophyllum*) and *buah kara* (*Ficus* spp.). The Ibans of Lambir in Sarawak reported a correlation between availability of fruits and appearance of hornbills in the ecosystem [33]. Barring figs that are found widely near human settlements, all other mentioned species are semi-domesticated ones planted close to human settlements. Although figs are known to comprise majority of the hornbill diet and possess essential nutrients [10, 61, 62], the contribution of non-fig fruits cannot be ignored [8]. Being frugivores, hornbills are known to be dependent on the availability of fruit trees [13, 27]. It could be said that the communities of Temburong through their cultural preference for fruit-bearing trees have contributed positively to the cause of hornbill conservation.

According to Iban TEK, the habitat preferences of Oriental Pied Hornbills and Asian Black Hornbills do not overlap. Although they are sub-species of the folk species *bruie*, the former is reported to be found mostly in wetlands such as mangroves, swamps and the nearby jungles, while Asian Black Hornbill prefers habitats other than wetlands. Mann [63] had recorded all seven species of hornbills except the Oriental Pied from the Belalong forests, lending credence to Iban TEK claim that they prefer the mangroves. Oriental Pied Hornbills have also been recorded from coastal townships and wetlands; studies have shown its preference for forests close to water bodies, thus agreeing with the Iban claim [64–67]. However, scientific knowledge shows that Asian Black and Oriental Pied Hornbills resort to resource sharing without competing with each other [68]. It should also be noted here that the mangrove forests of Temburong are known to comprise of relatively large trees [53], which could provide suitable nesting sites.

The Wreathed and Wrinkled Hornbills prefer riverine section of the Ulu Temburong. This habitat preference is corroborated by their preference for the Giant River Frogs (*Limnonectes leporinus*) that are found in streams and riparian forests of Ulu Temburong [50]. The recognition of ‘first come first serve’ rule in the right to hunt the Giant River Frog provides a glimpse into the existence of an Iban ‘code of hunting’. The Ibans recognise a hierarchy among the hornbills at the dietary and spatial levels, in the order of Rhinoceros > Helmeted Hornbill > Wrinkled and Wreathed Hornbills, followed by the rest. Both these Rhinoceros and Helmeted hornbills are known for their heavy dependence on fig for dietary purposes, and studies have shown that the fig species consumed by these species overlap significantly [27]. However, contrary to the Iban TEK, existing scientific records speculate the domination of helmeted hornbills over the rhinoceros hornbill owing to advantage of body size [10, 27]. It is possible that the Iban TEK in this context is unique to the locality, as there is indication of behavioural diversity in the scientific literature. For instance, in Sumatra, these two species share resources without competing and any expression of territoriality could only be facultative, arising when resources are limiting [13].

#### Reproductive aspects

The coinciding of hornbill breeding season with the fruiting season, the preference of Oriental Pied Hornbill for short trees and the group living nature of Bushy-crested Hornbills have been recorded in literature [11, 13, 66]. Likewise, the Iban TEK reporting high nest-site fidelity (philopatry) in all species has also been recorded in literature [69]. This shows the complementary nature of Iban TEK to existing scientific knowledge.

The Iban TEK on using the life stages of a seedling to index the growth phase of the hatchlings offers an extremely non-intrusive way to know the stage of the hornbill hatchling in the cavity. Indigenous communities are known to employ various indicators for temporal indexicalisation [70] to understand and manage their resources [71]. Perhaps, this unique temporal knowledge of the Ibans could have developed in the past when hornbill chicks were poached. Bartels and Bartels [72] describe the excitement of discovering young *Amorphophallus* growing from the expelled food beneath the nesting cavity of *Anthracocerós malayanus*. They also deduce the hatching of hornbill chicks from the presence of egg shells below the nest. Today, live filming of hornbills inside their cavity using non-intrusive, but expensive cameras is possible [73]. Yet, for conservation projects, the Iban TEK offers an equally precise, non-intrusive and affordable way to monitor hornbill chicks. Similar examples of extensive TEK on nesting phenology and reproductive behaviour of species have been also reported from species such as Tuatara, Turtles, and Seahorses [46, 74, 75]. Reduction of nestling provisions and desertion as claimed by the specialists has been reported in Zebra Finch [76], Bluebirds [77], etc., as strategies to enforce genetic monogamy. However, studies involving long-term observation of hornbills have failed to detect such desertion [78]. Iban ethnoecology recognises partner desertion as a strategy by male hornbills to enforce social monogamy, a claim that could further our understanding of hornbill reproduction.

#### Abundance, trends and threats

The population of hornbills increasing in Temburong is contrary to their global trend where populations have been constantly decreasing, with the exception of the Oriental Pied Hornbills [22]. Banning of shotguns and the migration of hornbills from Sarawak to Brunei are cited as the two major reasons for this phenomenon. The prohibition of possession and use of firearms and explosives follows the proclamation of emergency under Section 83 of the constitution following the rebellion period in the British protectorate of Brunei in 1962 [79]. Shotguns are reported to be one of major factors leading to excessive hunting of hornbills in Borneo [19], as they increase the efficiency and intensity of hunting [23, 25]. Hornbills are also protected as per Brunei’s Wild Life Protection Act of 1984 (revised edition), and the hunting/poaching of the protected birds is a serious offence. Clearly, the ban on shotguns and the protected status accorded to these birds have contributed to the increase in population. This study confirms the prediction of Mittermeier [80] that Brunei’s conservation policies would make an excellent example for wildlife conservation in the entire Southeast Asia.

The second reason cited by the elders for increase in hornbill population is the massive deforestation and logging activities in the neighbouring Malaysian state of Sarawak that has driven the birds to Temburong. This is corroborated by the claim of the Iban people in Lambir that reduction in fruit trees have led to fall in number of hornbills [33]. Studies from Malaysian Borneo indicate a negative relationship between industrial plantations and hornbill abundance [81]. The impact of logging on hornbill abundance on the other hand appears to be dependent on the intensity and nature of logging practised [14], and not logging per se. Studies report reduction in hornbill numbers due to logging [20, 82]. There are also studies reporting abundance of hornbill species in logged forests [14, 66]. The emerging consensus is that logging would lead to reduction in hornbill species if there is a reduction in fruit-bearing and nest-worthy trees [82, 83]. The positive correlation between abundance of fruits and hornbill population is well understood [8, 10], and it is possible that loss of fruiting trees in the neighbouring Malaysian Borneo due to logging and industrial plantations have led to the migration of hornbills to Temburong. While there is evidence to show that logged forests are also important for the conservation of hornbills [14, 16], studies on the role of fruit trees in orchards and home gardens in sustaining hornbills are lacking, though studies do record hornbills frequenting human settlements in the absence of hunting threats [84]. Our study indicates that the fruit trees in the human vicinity play a complementary role to the fruiting trees of the forests in sustaining hornbill populations.

### Current status of Iban TEK and its implications for conservation and ecotourism

Results of the semi-structured interviews show that the younger generation are not adept in TEK related to hornbills. Most of the respondents could only name *bruie* and *kenyalang*, indicating that their TEK is limited to the species they could spot in the vicinity of their households. Interaction with species is important for accumulating TEK on them, as understood from the case of Maori knowledge of Tuatara [74]. However, comparing this scenario with that of the elders, we understand that their TEK status is not much different from the younger generation. Only three members of the elder generation could list and identify all seven folk species while most of them could only name *kenyalang*, *kekalau* and *bruie*. Scholars have indicated the erosion of traditional knowledge, language and culture happening with the Iban people of Brunei and the larger Borneo [85]. Ellen and Bernstein [86], while discussing the cultural changes in Brunei, observe that the shift towards wage-based economy has resulted in urban drift, depopulation of longhouses and loss of forest-related knowledge in Brunei’s indigenous communities. According to them, such a change is coupled with an attitudinal change towards forests with forest-based livelihoods and knowledge considered as less important. TEK and indigenous languages are considered as inextricably linked to each other [87]. Sercombe (1999), while discussing the status of Iban language in Brunei, concludes that the language is largely intact. The findings of Coluzzi [57] also reflects the same, but indicates the beginning of a language shift towards the national language Malay which is of concern from the language point of view. Perhaps, the declining status of Iban TEK mirrors a decline of the Iban language. The silver lining emerging from the study is that the specialists have proven to be repositories of Iban TEK. It is noteworthy that they are actively involved in transmitting the TEK to the next generation on the field. Without these specialists, the Iban TEK would be lost forever as feared by Jarviie and Perumal [85].

Both hunting and traditional agriculture are key factors deciding acquisition and maintenance of Iban TEK. The impact of subsistence hunting on wildlife population and its relevance in biodiversity conservation will remain a widely debated topic in conservation science [23, 88]. The practice of hunting of hornbills for subsistence purposes in Borneo is as old as the very history of *Homo sapiens* in the island [89]. However, the scale, motive and technology of hunting in the region have changed since then [19, 23, 90]. At present, we do not have empirical data to speak in favour or against subsistence hunting in Temburong, though we know that Iban hunters are a minority, and hunting happens for subsistence purposes alone. We also know that the Iban hunters and agriculturists are living repositories of TEK and it is important to keep the in-field TEK transmission alive. For TEK on hornbills to exist, it is vital for the species to survive as it has been known that loss of species would also lead to loss of TEK [91]. Ecotourism and national park management are two immediate areas where the Iban TEK can be applied. Both ecotourism and co-management ventures involving local communities are known to keep intergenerational transmission of TEK alive [92–94]. However, at present, Ibans are able to undertake only low paid jobs in the tourism sector such as boat men and tour guides. Although being a tour guide is an opportunity to apply TEK, the emphasis unfortunately is on the scientific knowledge of hornbills and not TEK. Interestingly, application of TEK in managing Ulu Temburong National Park, or its co-management, was never suggested, indicating that the park is considered as a formal structure with no potential for incorporating TEK. Ahmad [95], while discussing benefits obtained by Ibans of Temburong through ecotourism, quotes his informant’s wish to expand his knowledge by interacting with the tourists as he finds his own knowledge insufficient. The statement of the tour guide whom we contacted indicates that Iban TEK is found to be lacking and not applied in ecotourism. Thus, we understand that Iban TEK is considered as deficient or inferior. Our study, however, shows that Iban TEK is not lost completely, but concentrated in the hands of specialists. An article by BizBrunei, dated 5 May 2018, reports that 12,000 Temburong tour packages were sold in 2017 alone, bulk of which were connected to the Ulu Temburong National Park [96]. We are tempted to say that there is a tremendous opportunity for the local communities including the Ibans to conserve their TEK and benefit by applying it in the ecotourism and conservation sectors.

## Conclusion

Our study adds to the growing body of literature on TEK [35], and the potential it holds in understanding Earth’s biodiversity. The Ibans recognise seven folk species of hornbills, with Asian Black Hornbill (*Anthracoceros malayanus*) and Oriental Pied Hornbill (*Anthracoceros albirostris*) considered as a single folk species. The Iban TEK provides information on the distribution, habitat, nesting and dietary preferences of all these hornbill species, complementing existing scientific knowledge on the species. However, Iban TEK is also able to provide additional information valuable to conservation. Examples are the importance of fruit trees in human vicinity in sustaining hornbill populations, hierarchical nature among hornbills and the non-intrusive technique of gauging growth stages of hornbill chicks through the seedlings growing beneath the nesting trees. The Iban TEK is also able to provide information on the conservation status of the species in the locality, and the factors responsible for the presumed increase in population that has belied global trends. By providing information on hornbill distribution, resources, reproduction and conservation status, Iban TEK proves to be of immense use in hornbill conservation and ecotourism ventures. However, much of the TEK is only possessed by Iban subsistence hunters and agriculturists who put it into use on the field and are also involved in its transmission. It is important to encourage active transmission of knowledge from these specialists through ecotourism and biodiversity co-management ventures. Such ventures will have to bring economic benefits to the community while respecting the cultural context of the Iban TEK and the role played by its custodians.

## Data Availability

The data supporting the conclusions of this article are included within the article.

## Abbreviations

Sp(p): Species
TEK: Traditional Ecological Knowledge
UTNP: Ulu Temburong National Park
